# Profiling the Secretion of Soluble Mediators by End Stage Osteoarthritis Synovial Tissue Explants Reveals a Reduced Responsiveness to an Inflammatory Trigger

**DOI:** 10.1371/journal.pone.0062634

**Published:** 2013-05-03

**Authors:** Lobke M. Gierman, Benno van El, Frits van der Ham, Angela Koudijs, Reinout Stoop, Jan H. Verheijen, Margreet Kloppenburg, Gerjo J. V. M. van Osch, Vedrana Stojanovic-Susulic, Tom W. J. Huizinga, Anne-Marie Zuurmond

**Affiliations:** 1 TNO, Leiden, The Netherlands; 2 Department of Rheumatology, Leiden University Medical Center, Leiden, The Netherlands; 3 Department of Orthopaedics and Department of Otorhinolaryngology, Erasmus MC, University Medical Center Rotterdam, Rotterdam, The Netherlands; 4 Pharmaceutical R&D, Janssen, A division of Johnson & Johnson, Malvern, Pennsylvania, United States of America; University of Leuven, Rega Institute, Belgium

## Abstract

**Objective:**

Evidence is accumulating that synovial tissue plays an active role in osteoarthritis (OA), however, exact understanding of its contribution is lacking. In order to further elucidate its role in the OA process, we aimed to identify the secretion pattern of soluble mediators by synovial tissue and to assess its ability to initiate cartilage degeneration.

**Methods:**

Synovial tissue explants (STEs) obtained from donors without history of OA (n = 8) or from end stage OA patients (n = 16) were cultured alone or together with bovine cartilage explants in the absence or presence of IL-1α. The secretion of 48 soluble mediators was measured and the effect on glycosaminoglycan (GAG) release and matrix metalloproteinase (MMP) activity was determined.

**Results:**

Normal and OA STEs secreted comparable levels of almost all measured soluble mediators. However, in the presence of IL-1α these mediators were less secreted by OA than by normal STEs of which 15 differed significantly (p<0.01). No effect of normal or OA STEs on GAG release from the cartilage explants was observed, and no differences in MMP activity between OA and normal STEs were detected.

**Conclusions:**

Unexpectedly, a comparable secretion profile of soluble mediators was found for OA and normal STEs while the reduced responsiveness of OA STEs to an inflammatory trigger indicates a different state of this tissue in OA patients. The effects could be the result of prolonged exposure to an inflammatory environment in OA development. Further understanding of the pro-inflammatory and inflammation resolving mechanisms during disease progression in synovial tissue may provide valuable targets for therapy in the future.

## Introduction

Osteoarthritis (OA) is one of the most frequently occurring rheumatic diseases. In Western populations OA is by far the most common form of joint disease, causing pain, loss of function and disability [1]. The main characteristic of the disease is progressive loss of articular cartilage, which is thought to be due to an imbalanced interplay between anabolic, anti-catabolic, anti- and pro-inflammatory and anti- and pro-apoptotic activities [2], [3]. Numerous risk factors for OA have been identified, however, the exact etiology, pathogenesis and progression of this disease have yet to be determined [4]. As a consequence of the limited understanding of the disease complexity, no disease modifying treatments are currently available. The only existing therapeutic strategies are primarily aimed at reducing pain and improving joint function.

Traditionally, research on knee OA has been focused on cartilage degradation. Nowadays, however, it is generally accepted that the entire joint organ including synovium, synovial fluid, bone and infrapatellar fat pad can contribute to the disease [5]. Inflammation, classically seen as a characteristic for rheumatoid arthritis (RA), has lately also been recognized for its role in OA development [6], [7]. Inflammation of the synovium (synovitis) has been shown to occur in a number of knee OA patients [8] and may produce proteases and cytokines that contribute to the disease. However, its role in the onset and progression in OA has yet to be elucidated.

It has been suggested that activated synovial macrophages might play a key role in the processes leading to synovial inflammation. This inflammation may act as a trigger for several symptoms of OA via release of soluble mediators by synovium, thus contributing to the breakdown of cartilage by promoting destruction and impairing the ability of repair [6]. The cytokines interleukin (IL)-1β and tumor necrosis factor (TNF)-α are likely to be one of those soluble mediators [9]. The understanding of the role of synovium and inflammation in the process of OA development could provide leads for new targets for OA treatment.

Based on published literature we hypothesized that synovium from OA patients is more inflamed than synovium of normal donors and therefore plays an active role in the breakdown of cartilage. To provide insight in this hypothesis, synovial tissue explants (STEs) of normal donors and OA patients were collected and analyzed for the spontaneous secretion of soluble mediators as well as their response to a pro-inflammatory trigger which was expected to be more increased in OA STEs than in normal STEs. We also used a complex *in vitro* co-culture model in which STEs were cultured together with cartilage explants in a Transwell system to assess the ability of STEs to initiate degeneration of healthy cartilage.

## Methods

### STEs Collection

Synovium of the knee was obtained from *post-mortem* material of 8 donors with macroscopically healthy cartilage and no history of OA (normal STE), or from material obtained during joint replacement surgery of 16 OA patients (OA STE) (Articular Engineering, Northbrook, USA). Human tissues were obtained according to legal and ethical requirements approved by the institutional review board of the University of Pennsylvania including anonymous informed written consent from the donor or nearest relative. Normal donors’ age ranged from 18–67 years and body mass index (BMI) from 19–37. OA donors’ age ranged from 43–70 and BMI from 18–47. Within 24 hours, synovial tissue was carefully excised from surrounding fat. Explants of 3 mm in size and a weight of 22±3.5 mg were placed in a 48-wells plate in Iscove’s Modified Dulbecco’s Medium (IMDM, Invitrogen, Paisley, UK) containing 10% v/v fetal calf serum (FBS) (GibcoBRL, Invitrogen) and 1% v/v of 10,000 units/ml Penicillin:10,000 units/ml streptomycin (Penstrep) (Biowhittaker, Verviers, Belgium). STEs were placed at 4°C until the start of the experiment. STEs were placed for 1 hour at 37°C in a humidified atmosphere of 5% CO_2_ in air before the start of the experiment.

### Immunohistochemistry of STEs

From each donor one STE was directly frozen in Tissue Tek O.C.T. compound (Sakura Finetek, Zoeterwoude, the Netherlands) and cut into 5 µm slices using a cryotome. Samples were thawed for 30 minutes and blocked with PBS containing 5% bovine serum albumin (BSA) (Sigma-Aldrich, St. Louis, USA). Sections were incubated overnight at 4°C with antibody CD68-biotin (mouse IgG2b 1∶750) (E-bioscience, San Diego, USA). The next day sections were washed with PBS and blocked for 5 minutes with peroxidase (Dako, Heverlee, Belgium). After a final wash step in PBS, sections were incubated with Novared for 10 minutes and counterstained with heamatoxylin. As a negative control an isotype matched control antibody was used (IgG2b-biotin, BD).

### STEs Culture

As a reflection of the whole synovium, for each condition 6 STEs from different anatomical locations in the joint per donor were cultured in a 24 wells plate in 700 µl Dulbecco’s Modified Eagle Medium (DMEM) (Invitrogen) supplemented with Insulin-transferrin-sodium selenite (ITS) (Roche Diagnostics, Basel, Switzerland), 1% v/v Penstrep, 1 mg/ml lactalbumin and 5 µg/ml vitamin C (Sigma-Aldrich) (serum free medium) and were incubated with or without 10 ng/ml IL-1α (Peprotech, Rocky Hill, USA). After 3 and 5 days 350 µl medium was refreshed and stored. After 7 days all medium was collected. Pooled supernatant samples were made representing the average of 6 different STEs per donor and the production over a time period of 7 days. All samples were stored at −80°C.

### Multiplex ELISA Assay

By using 2 commercial kits (42- human cytokine-plex and 11-human adipokine-plex, catalogue number: MPXHCYTO60KPMX42 and HADCYT-61K-11, Millipore, Billerica, USA) a wide panel of soluble mediators was measured in pooled supernatants of the STEs cultured alone. These assays were performed with Luminex xMap Technology (Qiagen, Billerica, USA) according to the manufacturer’s instructions. Liquichip analyzer software (Qiagen) was used for data analysis.

### Preparation of Cartilage Explants

Due to the availability and heterogeneity of human cartilage explants, standardization for *in vitro* models is difficult and therefore we used bovine cartilage explants [10]–[12]. Cartilage from the metacarpophanlangeal joints of 6 months old calves was obtained on the day of slaughter. Permission of the slaughterhouse (Ton Boer en zn., Nieuwerkerk a/d IJssel, the Netherlands) to use these joints in this experiment was given. Joints were aseptically opened and cartilage explants were obtained by using a biopsy punch of 4 mm. The cartilage explants were cultured in serum free medium overnight at 37°C in a humidified atmosphere of 5% CO_2_ in air.

### STE-cartilage Co-culture

Co-culturing of cartilage explants and STEs was performed in a 24-wells polycarbonate Transwell system with a pore size of 0.4 µm (Corning Incorporated, NY, USA). Each well contained a cartilage punch in the lower compartment and a STE in the upper compartment in 700 µl serum free medium with or without 10 ng/ml human IL-1α. Six STEs per donor per condition were used. As a control, cartilage was cultured alone. After 3 and 5 days 350 µl medium was refreshed and stored. After 7 days all medium was collected. Pooled supernatant samples were made representing the average of 6 different STEs per donor and the production over a time period of 7 days. All samples were stored at −80°C.

### Analysis of Glycosaminoglycans (GAGs)

Cartilage explants were digested 24 hours at 56°C in 3% v/v papain from papaya latex, 5 mM cysteine HCL, 50 mM EDTA, and 0.1 M sodium acetate (pH 5.53) (Sigma-Aldrich). The amount of glycosaminoglycans (GAGs), reflecting the amount of proteoglycans, was determined in cartilage explants as well as in culture medium using a commercial kit (Biocolor Ltd, Belfast, N. Ireland). The total GAG content was calculated by summing the amount of GAGs in the cartilage explant and the culture medium. Proteoglycan degradation was expressed as the percentage of GAG release into the medium compared to the total GAG content.

### Analysis of Matrix Metalloproteinase (MMP) Activity

Secreted MMP activity was assessed in the culture medium using a fluorogenic substrate as described previously [13]. The secreted MMP activity was calculated by determining the difference in substrate conversion in the presence or absence of MMP inhibitor BB94 (10 µM). This approach detects only MMP-mediated substrate conversion and reflects the MMP activity in the culture conditions.

### Data Analysis

Statistical analyses were performed using SPSS software version 17.0. Individual differences were tested by non-parametric Mann-Whitney tests. For comparisons of normal and OA STEs in the co-culture conditions, the GAG release of the cartilage explants were subtracted from the co-culture condition to correct for differences in bovine donors (Δ). Differences were considered statistically significant at p<0.01 for the Multiplex Elisa data. This cut-off was chosen to reduce the chance of false-positives, since a large number of variables were tested in a relative small number of donors due to the uniqueness of this material. For GAG release and MMP activity significance levels were set at p<0.05.

## Results

### Secretion of Soluble Mediators by STEs

**Figure 1:**
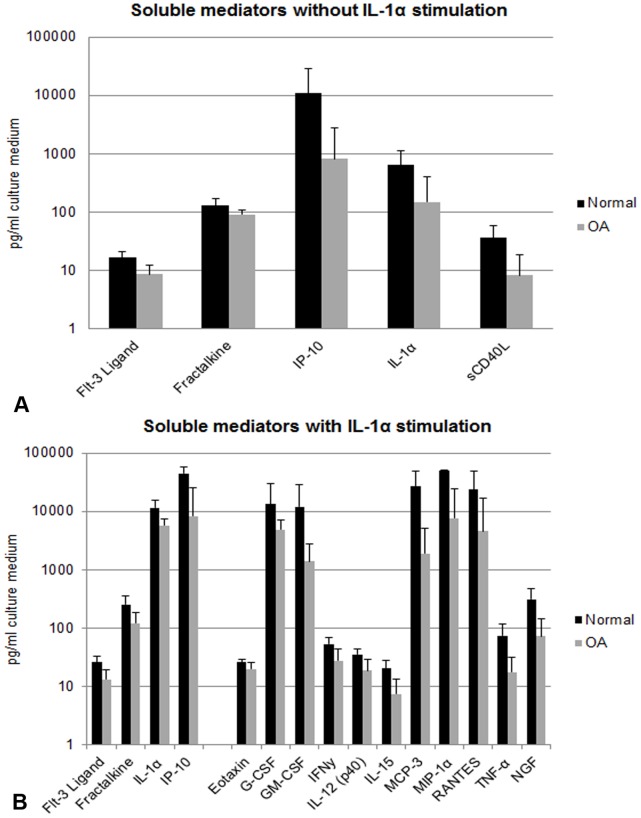
Soluble mediator secretion by normal and OA synovial tissue explants (STEs) with or without IL-1α. Graphs demonstrate all soluble mediators which were significantly (p<0.01) different between **A.** normal (black bars) and OA (grey bars) STEs and **B.** normal and OA STEs under pro-inflammatory (IL-1α) conditions. Data were obtained from pooled supernatants representing the average of 6 STEs per donor and the production in a time period of 7 days. Data are plotted on a log scale. Bars indicate mean concentration (pg/ml culture medium) ± SD.

To assess differences between normal and OA STEs 48 soluble mediators were measured in the pooled supernatants. Without IL-1α stimulation, only 5 soluble mediators were secreted at significantly different levels (p<0.01). Remarkably, these mediators were excreted at a lower level by OA STEs than normal STEs (Figure 1A).

Under pro-inflammatory (IL-1α-stimulated) conditions 15 of the measured mediators were significantly less secreted by OA STEs than by normal STEs (p<0.01). Of these 15 mediators, 4 were also significantly different in the absence of IL-1α (Figure 1B). IL-13, IL-17, IL-2, IL-4, IL-9, PDGF-AB, TGFα, TNF-β and IL-12p70 could not be detected in either condition.

**Figure 2:**
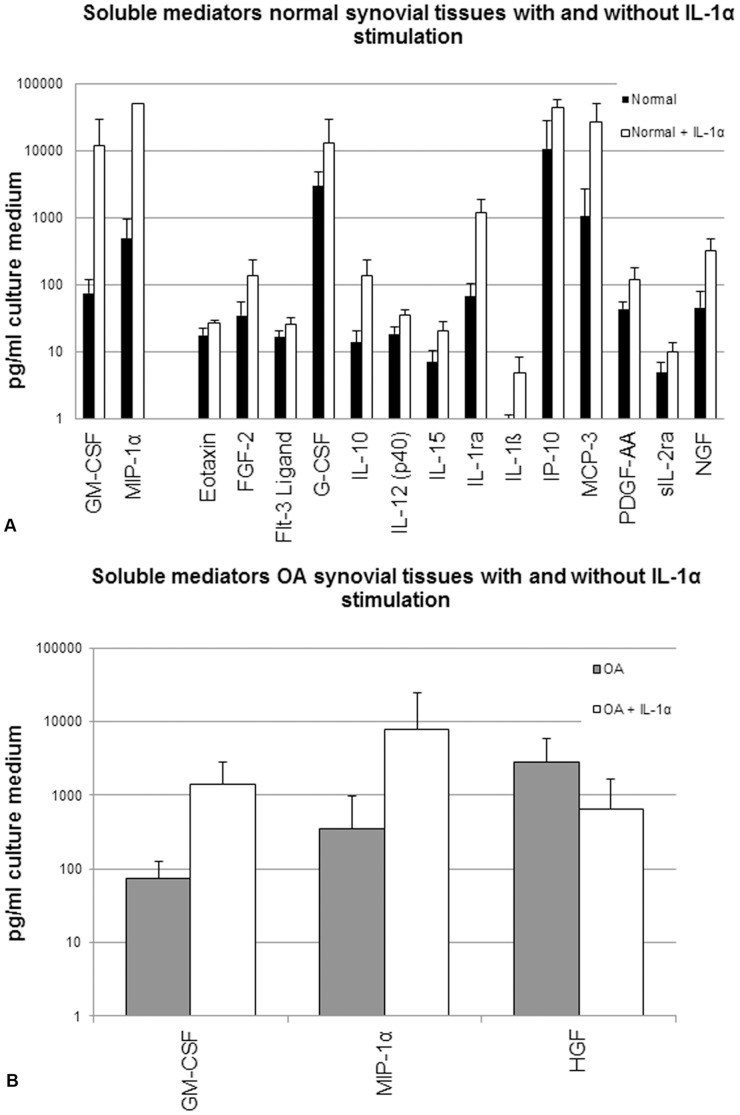
Soluble mediator secretion by non-stimulated and stimulated synovial tissue explants (STEs) from normal and OA donors. Graphs demonstrate the absolute levels of all soluble mediators, which were significantly (p<0.01) different between **A.** non-stimulated (black bars) and IL-1α-stimulated (white bars) normal STEs and **B.** non-stimulated (grey bars) and IL-1α-stimulated (white bars) OA STEs. Data were obtained from pooled supernatants representing the average of 6 STEs per donor and the production in a time period of 7 days. Data are plotted on a log scale. Bars indicate mean concentration (pg/ml culture medium) ± SD.

To assess the response to an inflammatory trigger of STEs from normal and OA donors, soluble mediator excretion levels were analyzed in both the non-stimulated and the IL-1α-stimulated condition. For normal STEs, 16 mediators increased significantly (p<0.01) after stimulation with IL-1α (Figure 2A). Unexpectedly, the STEs obtained from OA donors secreted only 3 mediators at a significantly different level after IL-1α stimulation (Figure 2B). An overview of all measured mediators in these supernatants can be consulted in table S1.

Immunohistochemistry on one STE from each donor showed well known characteristics of OA such as hyperplasia as observed by an increase in cell number and thickening of the synovial layer. CD68+ cells were present in both OA and normal STEs, indicating the presence of synovial macrophages during the culture period (Figure S1).

### Co-culture Effects on GAG-release and MMP-activity in Cartilage Explants

**Figure 3:**
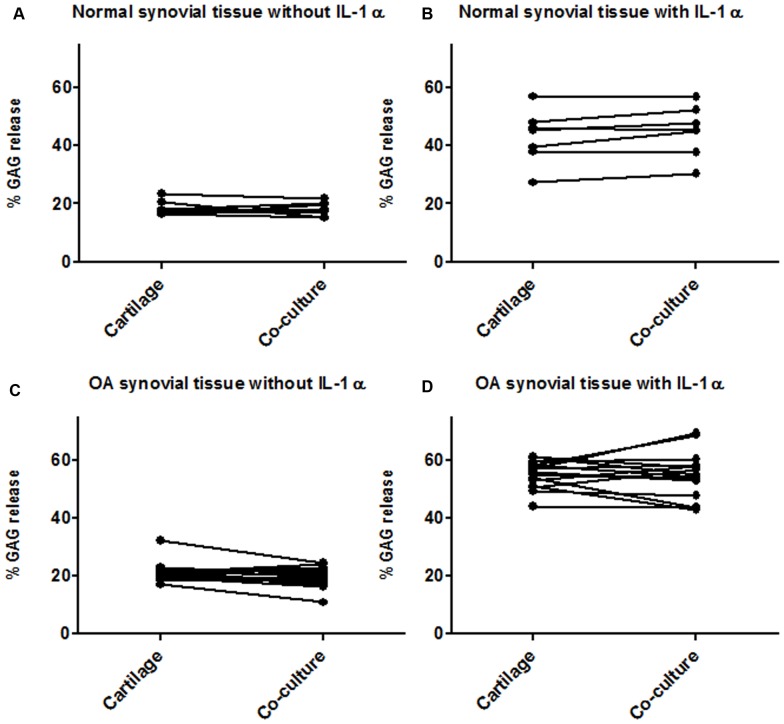
Proteoglycan degradation of healthy cartilage explants cultured alone or together with synovial tissue explants (STEs). Proteoglycan degradation was expressed as the percentage of glycosaminoglycans (GAG) released into the medium during 7 days of culture of cartilage alone or co-cultured together with normal (**A, B**) or OA (**C, D**) STEs without (**A, C**) or with (**B, D**) IL-1α stimulation. Each line represents an individual donor and connects the % GAG release of cartilage alone (left) with the matching co-culture condition of cartilage together with STEs (right). There were no significant differences between cartilage cultured alone or co-cultured with normal or OA STEs.

Based on multiplex analysis we questioned if there was also a different effect of normal and OA STEs on cartilage degradation. Hereto, we used an *in vitro* co-culture model in which cartilage explants were cultured together with STEs. Co-culturing with normal or OA STEs did not induce additional GAG release above the basal release of cartilage explants in non- and IL-1α-stimulated culture conditions (Figure 3). IL-1α stimulation led to significantly more GAG release in all culture conditions (table 1). When comparing absolute values, normal and OA donors differed significantly, however, this was due to a significant difference in basal release of the cartilage explants and not for additional GAG release induced by STEs (indicated in table 1 by the calculated delta (Δ)).

**Table 1:** Percentage GAG release from cartilage explants cultured alone or together with STEs under non-stimulated and IL-1α-stimulated conditions.

Condition	% GAG release				P-values			
	Normal	Normal(IL-1α)	OA	OA (IL-1α)	Normal vsNormal (IL-1α)	OA vs OA(IL-1α)	Normal vs OA	Normal (IL-1α)vs OA (IL-1α)
**Cartilage**	18.6 (2.6)	43.1 (9.3)	21.5 (3.4)	54.9 (4.7)	0.002	0.000	0.026	0.005
**STE+cartilage**	18.2 (2.5)	45.1 (8.8)	19.6 (3.5)	54.8 (8.1)	0.002	0.000	0.245	0.032
**Δ STE+cartilage**	−0.32 (2.9)	1.95 (2.5)	−1.9 (3.0)	−0.15 (6.4)	0.180	0.576	0.245	0.267

%GAG release is indicated as mean (SD).

Δ STE+cartilage: corrected value for GAG release in the cartilage explant.

**Figure 4:**
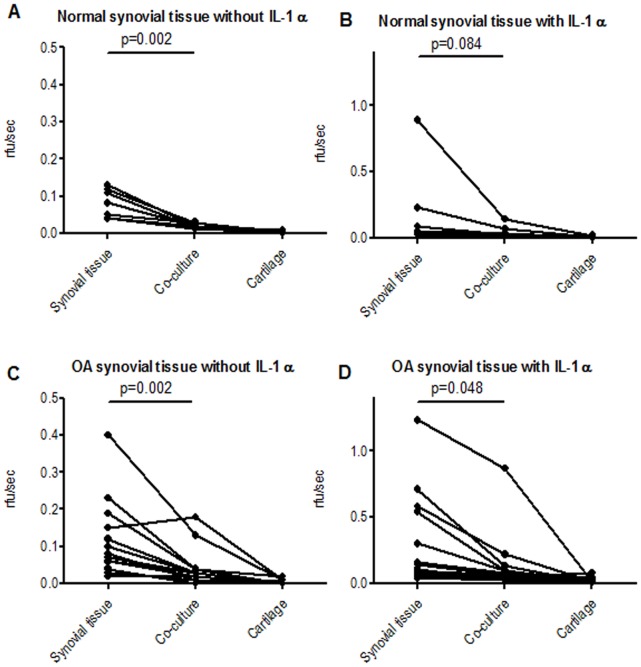
Secreted MMP activity of STEs and cartilage explants cultured alone or together. After 7 days, the MMP-activity (rfu/sec) was determined (in culture medium) of normal (**A, B**) and OA (**C, D**) STEs and healthy cartilage explants, cultured alone or together, without (**A, C**) or with (**B, D**) IL-1α stimulation. Each line represents an individual donor and demonstrates the activity of STEs alone (left), STEs together with cartilage explants (middle) or cartilage explants cultured alone (right). The MMP activity in the medium of STEs cultured alone was higher, compared to the MMP activity in the co-culture conditions. Significance levels are indicated.

To assess for collagen degrading enzymes, MMP activity was measured in the different culture conditions. Remarkably, the MMP activity in the medium of non-stimulated normal and OA STEs cultured alone was significantly higher than in the co-cultures (p<0.01, Figure 4A,C). This was also observed for the IL-1α-stimulated OA condition (Figure 4D; p<0.05). A trend was observed in the IL-1α-stimulated normal condition (Figure 4B; p<0.1). IL-1α stimulation did not led to significantly more MMP activity when normal or OA STE was cultured alone (table 2). IL-1α stimulation yielded significantly more MMP activity in the co-cultures with OA STE while this was not the case for normal STE culture conditions. This is probably due to the fact that bovine cartilage donors used in the OA STE conditions were more prone to IL-1α stimulation with respect to MMP activity than those used in normal STE conditions (table 2).

**Table 2:** Secreted MMP activity (rfu/sec) by STE and cartilage alone and by the co-culture of STE and cartilage under non-stimulated and Il-1α-stimulated conditions.

Condition	Secreted MMP-activity (rfu/sec)				P-values			
	Normal	Normal(IL-1α)	OA	OA (IL-1α)	Normal vs Normal (IL-1α)	OA vs OA(IL-1α)	Normal vs OA	Normal (IL-1α)vs OA (IL-1α)
**Cartilage**	0.003 (0.003)	0.009 (0.007)	0.003 (0.006)	0.023 (0.018)	0.170	0.000	0.232	0.007
**STE**	0.081 (0.039)	0.196 (0.314)	0.115 (0.098)	0.280 (0.343)	0.898	0.245	0.596	0.244
**STE+cartilage**	0.021 (0.006)	0.047 (0.045)	0.042 (0.048)	0.126 (0.212)	0.174	0.004	0.121	0.061

Secreted MMP activity is indicated as mean (SD).

## Discussion

Increasing evidence indicates that inflammation of the synovium is a feature of OA associated with the progression of disease [6]. It remains, however, unclear in which way alterations in the synovium contribute to degenerative processes in the cartilage. By measuring the secretion of soluble mediators by the synovium and investigate its effect on cartilage break-down, we hoped to identify new mechanisms in OA and to provide new targets to intervene in the disease process. Unexpectedly, STEs from end stage OA patients showed comparable secretion levels as normal STEs for a wide panel of soluble mediators, suggesting that the inflammatory state of end stage OA-derived synovium is not different from normal. Differences between normal and end-stage OA STEs appeared after stimulation with IL-1α showing a diminished responsiveness of OA compared to normal STEs. Furthermore, STEs derived from end stage OA patients did not induce more GAG release from healthy cartilage explants or displayed more MMP activity than normal STEs in the presence or absence of a pro-inflammatory trigger. This suggests that synovium from end stage OA patients is not, or no longer capable of initiating cartilage degradation.

With regard to OA, the pro-inflammatory cytokines IL-1β and TNFα are most frequently studied and detected. Furthermore IFN-γ, IL-2, IL-4, IL-6, IL-8, TGFβ and IL-10 have received attention as important cytokines in the OA process [14]–[17]. Of these soluble mediators all, except IL-2, were secreted by STEs. Unexpectedly, IL-1α, one of the key cytokines in the pathogenesis of OA [18], was significantly less secreted by end stage OA STEs than by normal STEs.

The unexpected effects in mediator release and on GAG release seen in our study could be explained by the state of the STEs. All OA STEs were obtained from OA patients undergoing joint replacement surgery and are therefore in the end stage of the disease. Originally, it was thought that the condition of the synovium was correlated with OA severity and occurred gradually through the disease process [19], [20]. However, Roemer et al. showed that baseline joint effusion and synovitis predicted the risk of cartilage loss after 30 months follow up [21]. This indicates an important role for inflammation in an earlier stage of OA. Furthermore, Benito et al. demonstrated that early OA synovium has more features of inflammation (greater cell infiltration and overexpression of inflammatory mediators) than late OA synovium [8]. In line with this, Ning et al. found a decreased expression of MMP-1, COX-2 and IL-1β expression in synovium depending upon the severity of OA [17]. These previous observations are in agreement with our result that end stage OA STEs display an excretion profile that is rather similar to that of normal controls. The occurrence of inflammation resolving mechanisms in end stage OA STEs due to exposure to inflammatory triggers earlier in life might explain this observation. This is substantiated by its diminished responsiveness to a pro-inflammatory trigger. Potential molecular mechanism would be the down regulation of many receptors and a decreased secretion of resolving mediators to counter balance chronic inflammation. The fact that MMP activity seemed to be down regulated when co-cultured with cartilage explants also contributes to this idea. Furthermore, end-stage OA STEs were not able to induce cartilage degeneration in a co-culture model which endorses the idea that synovium is not capable of initiating cartilage degradation. However, it does not rule out the possibility that it plays a role in progression and aggravation of the cartilage destruction process in OA, since we have not tested OA and normal STEs on OA-derived cartilage.

Beekhuizen et al. have demonstrated in a comparable *in vitro* co-culture model with matched OA cartilage and OA STEs an effect on GAG production, but not on GAG release [22]. Due to our design, we were not able to determine GAG synthesis by, for example, the rate of sulphate incorporation. However, based on total GAG content (data not shown) we do not expect an effect of OA or normal STEs on GAG synthesis.

Remarkably, it seems that IL-1α is not involved in the activation of STEs leading to cartilage destruction, since no increase in GAG release was observed even though numerous soluble mediators were elevated. The effect of the IL-1α trigger on GAG release and MMP activity can be subscribed to its direct effect on chondrocytes without any contribution of IL-1α- stimulated STEs. However, other explanations could be that IL-1α overrules the effect of the secreted soluble mediators or not all human mediators excreted by STEs cross-react with bovine receptors.

Minor points of the study need to be addressed. As the excretion of soluble mediators is not corrected for the amount of cells, the absolute levels can be discussed. To reach feasible standardization in this experiment the results were based on multiple explants per donor with comparable weights. Furthermore, the presence of small pieces of other tissues as well as the injury response at collection cannot be fully excluded. At last, we cannot exclude that hypoxia in *post mortem* obtained normal STEs influenced the outcome of the study although hypoxia inducible factor-driven VEGF levels were not different between normal and OA STEs [23], [24]. Due to the fact that the procedures for OA and normal STEs were standardized we expect that the observed differences include comparable limitations.

In summary, we found the excretion profile of OA STEs to be comparable to that of normal donors. This is in contrast to what we had anticipated, namely more inflamed tissue with elevated secretion of pro-inflammatory mediators and MMPs. As such it is understandable that no contribution of the OA STEs was observed on cartilage destruction in the co-culture model. The responsiveness of the STEs to a pro-inflammatory trigger, however, indicates that there are differences in the state of the tissue between OA and normal donors. It suggests that end stage OA STEs are less sensitive to inflammatory stimulation which might be due to prolonged exposure to an inflammatory environment during disease progression. However, this does not rule out the involvement of STEs in an earlier phase of OA development. Further understanding of the pro-inflammatory and inflammation resolving mechanisms during disease progression in synovium may provide valuable targets for therapy in the future.

## Supporting Information

Figure S1 **Immunohistochemical staining for CD68+ cells in representative synovial tissue explants.**
(DOCX)

Table S1 **Soluble mediator secretion by normal and OA synovial tissue explants.**
(DOCX)
