# Supplementary material for: Profiling the Secretion of Soluble Mediators by End Stage Osteoarthritis Synovial Tissue Explants Reveals a Reduced Responsiveness to an Inflammatory Trigger
Source: PLoS One. 2013 May 3;8(5):e62634. doi: 10.1371/journal.pone.0062634 (PMC3643929; doi:10.1371/journal.pone.0062634)
Supplement: Figure S1 — Immunohistochemical staining for CD68+ cells in representative synovial tissue explants. (DOCX) [file pone.0062634.s001.docx]

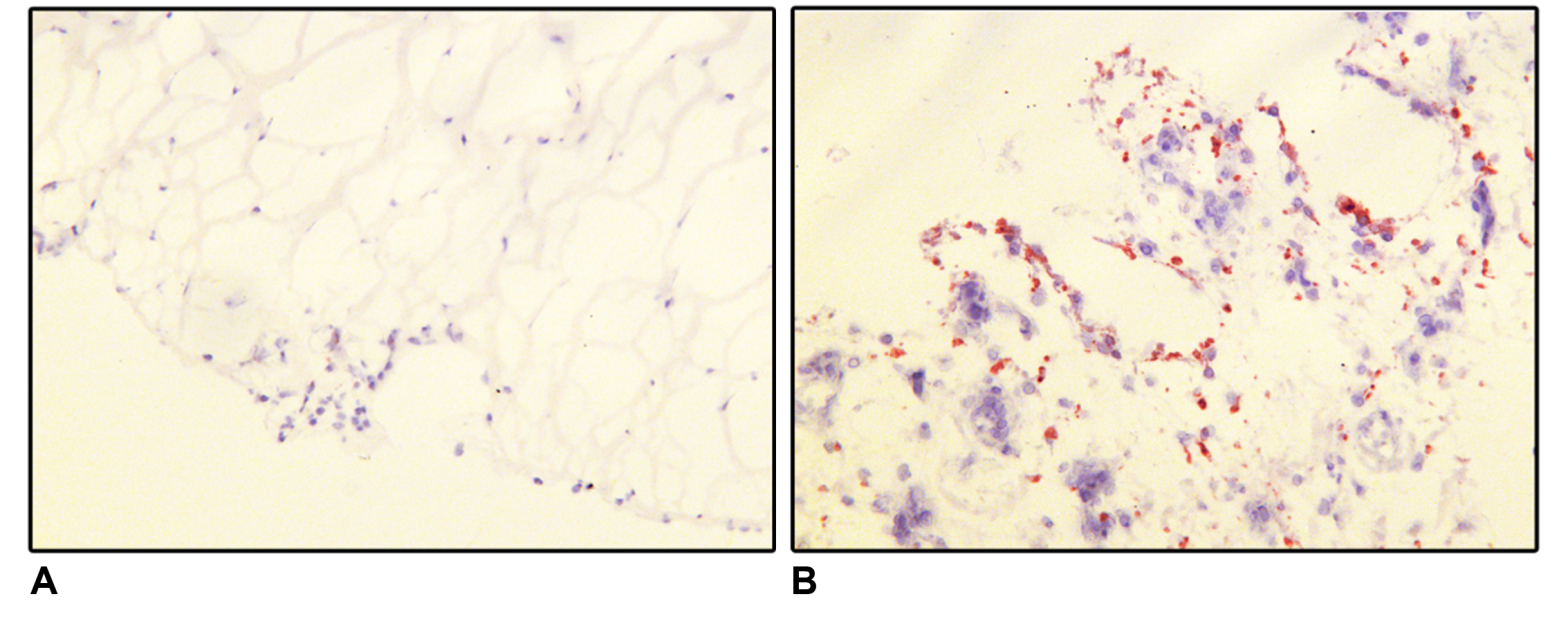


**Figure S2** Immunohistochemical staining for CD68+ cells in representative synovial tissue explants directly after acquiring. **A.** normal donor and **B.** OA donor. Magnification 20x
