# Supplementary material for: Profiling the Secretion of Soluble Mediators by End Stage Osteoarthritis Synovial Tissue Explants Reveals a Reduced Responsiveness to an Inflammatory Trigger
Source: PLoS One. 2013 May 3;8(5):e62634. doi: 10.1371/journal.pone.0062634 (PMC3643929; doi:10.1371/journal.pone.0062634)
Supplement: Table S1 — Soluble mediator secretion by normal and OA synovial tissue explants. (DOCX) [file pone.0062634.s002.docx]

|  | **Mean (SD) Normal** | **Mean (SD)**  **OA** | **Mean (SD) Normal (IL-1α)** | **Mean (SD)**  **OA (IL-1α)** | **p-value Normal vs OA** | **p-value Normal (IL-1α) vs OA (IL-1α)** | **p-value Normal vs Normal (IL-1α)** | **p-value**  **OA vs OA (IL-1α)** |
| --- | --- | --- | --- | --- | --- | --- | --- | --- |
| **EGF** | 2.1 (2.7) | 1.1(1.6) | 2.6 (3.4) | 1.1(1.5) | 0.56 | 0.46 | 0.83 | 0.93 |
| **Eotaxin** | 17.8(4.5) | 14.0(7.0) | 26.4(3.1) | 20.0(5.5) | 0.10 | **0.005 | **0.002 | *0.01 |
| **FGF-2** | 34.7(19.8) | 28.5(20.0) | 137.0(103.1) | 52.0(31.8) | 0.35 | **.016 | **0.009 | *0.011 |
| **Flt-3 Ligand** | 16.6(4.2) | 8.7(3.8) | 26.4(6.6) | 13.3(6.2) | **0.001 | ***0.001 | **0.007 | *0.015 |
| **Fractalkine** | 128.7(42.5) | 90.9 (20.0) | 253.5(111.5) | 121.6(62.9) | **0.005 | **0.006 | *0.018 | *0.026 |
| **G-CSF** | 2946.2(1944.8) | 3255.7(2656.4) | 13218.7(16253.7) | 4845.4(2343.5) | 0.84 | **0.008 | **0.003 | 0.12 |
| **GM-CSF** | 76.2(42.7) | 74.6 (54.0) | 12072.6(16901.8) | 1401.2(1423.8) | 0.74 | ***0.001 | **0.002 | ***0.001 |
| **GRO** | 21428.6(19518) | 29079.7 (21731.6) | 27142.9(21380.9) | 15407.9(17573.6) | 0.60 | 0.11 | 0.59 | 0.07 |
| **IFNa2** | 52.0(19.9) | 40.2(15.0) | 58.7 (20.4) | 42.9 (24.5) | 0.14 | 0.19 | 0.48 | 0.76 |
| **IFNy** | 34.7(17.0) | 30.2 (14.6) | 53.3(15.7) | 28.0 (16.8) | 0.64 | **0.005 | *0.048 | 0.84 |
| **IL-10** | 14.0(6.7) | 17.0 (12.7) | 138.3(98.4) | 69.5(80.1) | 0.71 | 0.06 | **0.009 | *0.019 |
| **IL-12 (p40)** | 18.8(5.0) | 14.1(5.2) | 35.5(7.8) | 18.8(10.6) | 0.06 | **0.003 | **0.002 | 0.21 |
| **IL-15** | 7.0(3.5) | 4.1(2.7) | 20.4(8.1) | 7.6(5.7) | 0.06 | **0.002 | **0.003 | 0.08 |
| **IL-1α** | 639.8(471.7) | 149.1(256.9) | 11528.7(4095.5) | 5744.9(1685.4) | **0.008 | **0.001 | **0.002 | ***0.001 |
| **IL-1ra** | 67.2(39.0) | 255.5 (414.4) | 1168.0(697.3) | 1059.1(1873.6) | 0.32 | 0.08 | **0.002 | 0.13 |
| **IL-1ß** | 0.5(0.6) | 2.0 (3.4) | 4.8(3.7) | 4.4(6.5) | 0.22 | 0.20 | **0.002 | 0.15 |
| **IL-3** | 4.9(6.2) | 1.1(2.2) | 4.5(6.9) | 0.6(1.0) | 0.27 | 0.51 | 0.94 | 0.81 |
| **IL-5** | 0.1(0.0) | 0.1(0.1) | 0.2(0.1) | 0.2(0.2) | 0.52 | 0.35 | 0.10 | 0.37 |
| **IL-6** | 25450.7(22964.7) | 20452.0(20574.9) | 25436.9(22977.3) | 8820.2(11234.0) | 0.36 | 0.07 | 0.89 | *0.092 |
| **IL-7** | 63.7(12.7) | 61.4(20.0) | 61.9(18.7) | 53.1(21.3) | 0.46 | 0.32 | 0.95 | 0.99 |
| **IL-8** | 24015.8(24309.1) | 24325.3(23387.5) | 17449.0(22238.5) | 9505.2(15879.5) | 0.78 | 0.35 | 0.47 | 0.07 |
| **IP-10** | 10792.7(17747.9) | 823.3(1949.0) | 44637.7(14187.2) | 8301.9(16614.8) | **0.003 | ***0.001 | **0.005 | *0.038 |
| **MCP-1** | 13106.1(16350.2) | 5940.7 (1750.2) | 7776.2(3328.6) | 6159.3(5842.8) | *0.045 | 0.14 | 0.41 | 0.49 |
| **MCP-3** | 1061.7(1612.9) | 378.8 (706.3) | 26633.4(22873.2) | 1884.8(3176.7) | *0.027 | **0.005 | **0.009 | 0.16 |
| **MDC** | 10.0(12.3) | 35.1(55.9) | 17.6(10.9) | 56.9(87.0) | 0.32 | 0.74 | 0.18 | 0.56 |
| **MIP-1α** | 489.9 (451.4) | 348.1(637.9) | 50000.0(0.0) | 7708.6(16573.8) | 0.11 | ***0.001 | **0.001 | **0.002 |
| **MIP-1ß** | 216.9(101.4) | 167.7(202.3) | 1013.4(820.3) | 410.1(386.8) | 0.29 | 0.08 | *0.013 | 0.15 |
| **PDGF-AA** | 43.5(12.6) | 122.0(107.8) | 122.1(54.1) | 231.8(233.7) | 0.07 | 0.74 | **0.002 | 0.20 |
| **RANTES** | 7924.8(18582.4) | 510.9(882.2) | 23564.6(24730.1) | 4586.5(12200.3) | *0.045 | **0.003 | *0.014 | *0.016 |
| **sCD40L** | 36.4(22.9) | 8.3(10.5) | 54.0(10.7) | 26.0 | **0.002 | *0.045 | 0.11 | *0.016 |
| **sIL-2Ra** | 4.9(2.2) | 3.9(2.0) | 10.2 (3.3) | 8.1 | 0.35 | 0.19 | **0.006 | *0.036 |
| **TNF-α** | 21.9(14.6) | 7.3(6.1) | 75.0(43.4) | 17.6 | *0.012 | **0.001 | 0.01 | *0.029 |
| **VEGF** | 251.5(202.1) | 343.4(421.0) | 775.4(523.3) | 690.7 | 0.84 | 0.59 | *0.018 | *0.046 |
| **Adiponectin** | 10322.5(5338.3) | 20991.6(9787.8) | 11991.8 (10521.7) | 20518.5 | *0.021 | 0.50 | 0.57 | 0.52 |
| **HGF** | 4120.3(2662.5) | 2791.5(3177.3) | 1004.8 (641.0) | 647.0 | 0.41 | 0.07 | 0.06 | **0.002 |
| **Leptin** | 20.7(24.3) | 92.3(181.3) | 45.6 (50.4) | 268.0 | 0.50 | 0.84 | 0.31 | 0.86 |
| **NGF** | 46.0 | 22.5(20.0) | 316.1(168.8) | 73.1 | 0.10 | ***0.001 | **0.004 | 0.06 |
| **PAI-1 Total** | 1242.6 | 891.5(486.6) | 1704.0 (578.9) | 1429.3 | 0.25 | 0.38 | 0.17 | 0.16 |
| **Resistin** | 16.9 | 33.9(61.6) | 405.6(1006.2) | 65.9 | 0.30 | 0.33 | 0.41 | 0.69 |

**Table S1.** Mean (SD) levels (pg/ml culture medium) of soluble mediators excreted by normal and OA synovial tissue explants without (Normal and OA) and with IL-1α stimulation (Normal (IL-1α) and OA (IL-1α)).

***p<0.001, **p<0.01, *p<0.05.
